# Pharmacological modulation of TSPO in microglia/macrophages and neurons in a chronic neurodegenerative model of prion disease

**DOI:** 10.1186/s12974-023-02769-y

**Published:** 2023-04-09

**Authors:** Marta Vicente-Rodríguez, Renzo Mancuso, Alba Peris-Yague, Camilla Simmons, NIMA Consortium, Dominika Wlazly, Amber Dickinson, Andy Foster, Clare Knight, Claire Leckey, Paul Morgan, Angharad Morgan, Caroline O’Hagan, Samuel Touchard, Shahid Khan, Phil Murphy, Christine Parker, Jai Patel, Jill Richardson, Paul Acton, Nigel Austin, Anindya Bhattacharya, Nick Carruthers, Peter de Boer, Wayne Drevets, John Isaac, Declan Jones, John Kemp, Hartmuth Kolb, Jeff Nye, Gayle Wittenberg, Gareth Barker, Anna Bogdanova, Heidi Byrom, Annamaria Cattaneo, Daniela Enache, Tony Gee, Caitlin Hastings, Melisa Kose, Giulia Lombardo, Nicole Mariani, Anna McLaughlin, Valeria Mondelli, Maria Nettis, Naghmeh Nikkheslat, Carmine Pariante, Karen Randall, Julia Schubert, Luca Sforzini, Hannah Sheridan, Nisha Singh, Vicky Van Loo, Mattia Veronese, Toby Wood, Courtney Worrell, Zuzanna Zajkowska, Brian Campbell, Jan Egebjerg, Hans Eriksson, Francois Gastambide, Karen Husted Adams, Ross Jeggo, Thomas Moeller, Bob Nelson, Niels Plath, Christian Thomsen, Jan Torleif Pederson, Stevin Zorn, Catherine Deith, Scott Farmer, John McClean, Andrew McPherson, Nagore Penandes, Paul Scouller, Murray Sutherland, Mary Jane Attenburrow, Jithen Benjamin, Helen Jones, Fran Mada, Akintayo Oladejo, Katy Smith, Rita Balice-Gordon, Brendon Binneman, James Duerr, Terence Fullerton, Veeru Goli, Zoe Hughes, Justin Piro, Tarek Samad, Jonathan Sporn, Liz Hoskins, Charmaine Kohn, Lauren Wilcock, Franklin Aigbirhio, Junaid Bhatti, Ed Bullmore, Sam Chamberlain, Marta Correia, Anna Crofts, Tim Fryer, Martin Graves, Alex Hatton, Manfred Kitzbichler, Mary-Ellen Lynall, Christina Maurice, Ciara O’Donnell, Linda Pointon, Peter St George Hyslop, Lorinda Turner, Petra Vertes, Barry Widmer, Guy Williams, Jonathan Cavanagh, Alison McColl, Robin Shaw, Erik Boddeke, Alison Baird, Stuart Clare, Phil Cowen, I-Shu Huang, Sam Hurley, Alejo Nevado-Holgado, Elena Ribe, Anviti Vyas, Laura Winchester, Madeleine Cleal, Diego Gomez-Nicola, Hugh Perry, Mara Cercignani, Charlotte Clarke, Alessandro Colasanti, Neil Harrison, Rosemary Murray, Jason O’Connor, Howard Mount, Diego Gómez-Nicola, V. Hugh Perry, Federico Turkheimer, Simon Lovestone, Christine A. Parker, Diana Cash

**Affiliations:** 1 Department of Neuroimaging, BRAIN Centre (Biomarker Research and Imaging for Neuroscience), Institute of Psychiatry, Psychology & Neuroscience, King’s College London, London, UK; 2 The Wellcome Trust Consortium for the Neuroimmunology of Mood Disorders and Alzheimer’s Disease (NIMA), London, UK; 3 Departamento de Ciencias Farmacéuticas y de la Salud, Facultad de Farmacia, Universidad San Pablo-CEU, CEU Universities, Madrid, Spain; 4 Microglia and Inflammation in Neurological Disorders (MIND) Lab, VIB Center for Molecular Neurology, VIB, Antwerp, Belgium; 5 Department of Biomedical Sciences, University of Antwerp, Antwerp, Belgium; 6 Biological Sciences, Southampton General Hospital, University of Southampton, Southampton, UK; 7 GlaxoSmithKline, Stevenage, London, UK; 8 Janssen Medical Ltd, High Wycombe, UK

**Keywords:** Astrocytes, CSF1R, ME7, Microglia, Neuroinflammation, Neurons, TSPO, Prion disease

## Abstract

Neuroinflammation is an important component of many neurodegenerative diseases, whether as a primary cause or a secondary outcome. For that reason, either as diagnostic tools or to monitor progression and/or pharmacological interventions, there is a need for robust biomarkers of neuroinflammation in the brain. Mitochondrial TSPO (18 kDa Translocator protein) is one of few available biomarkers of neuroinflammation for which there are clinically available PET imaging agents. In this study, we further characterised neuroinflammation in a mouse model of prion-induced chronic neurodegeneration (ME7) including a pharmacological intervention via a CSF1R inhibitor. This was achieved by autoradiographic binding of the second-generation TSPO tracer, [3H]PBR28, along with a more comprehensive examination of the cellular contributors to the TSPO signal changes by immunohistochemistry. We observed regional increases of TSPO in the ME7 mouse brains, particularly in the hippocampus, cortex and thalamus. This increased TSPO signal was detected in the cells of microglia/macrophage lineage as well as in astrocytes, endothelial cells and neurons. Importantly, we show that the selective CSF1R inhibitor, JNJ-40346527 (JNJ527), attenuated the disease-dependent increase in TSPO signal, particularly in the dentate gyrus of the hippocampus, where JNJ527 attenuated the number of Iba1+ microglia and neurons, but not GFAP+ astrocytes or endothelial cells. These findings suggest that [3H]PBR28 quantitative autoradiography in combination with immunohistochemistry are important translational tools for detecting and quantifying neuroinflammation, and its treatments, in neurodegenerative disease. Furthermore, we demonstrate that although TSPO overexpression in the ME7 brains was driven by various cell types, the therapeutic effect of the CSF1R inhibitor was primarily to modulate TSPO expression in microglia and neurons, which identifies an important route of biological action of this particular CSF1R inhibitor and provides an example of a cell-specific effect of this type of therapeutic agent on the neuroinflammatory process.

**Supplementary Information:**

The online version contains supplementary material available at 10.1186/s12974-023-02769-y.

## Introduction

Many neurodegenerative diseases, such as Alzheimer’s, Parkinson’s and prion diseases, share related pathogenetic mechanisms involving aggregation and deposition of misfolded proteins, leading to progressive neuronal degeneration of the central nervous system (CNS). Another common pathological hallmark of neurodegeneration appears to be chronic neuroinflammation, mediated by disease-associated activation of microglia and astrocytes [1]. Attempting to modulate and attenuate these inflammatory processes has become an important focus of clinical neuroscience research.

Mitochondrial TSPO (18 kDa Translocator protein) is one of the few available biomarkers of neuroinflammation for which there are clinically available positron emission tomography (PET) imaging agents. TSPO is an outer mitochondrial membrane protein whose role is not completely elucidated [2–5], but is purported to be implicated in a variety of cellular and mitochondrial functions, including biosynthesis of steroids and haeme, porphyrin transport, apoptosis, cell proliferation, and neuromodulation [6, 7]. Overall there is an emerging consensus that the primary role of TSPO may be in regulation of cellular metabolic processes [8], many of which are also recruited during inflammation [9]. Accordingly, using PET imaging, increased TSPO signal has been observed in a range of human neurodegenerative diseases with an inflammatory component, such as simian immunodeficiency virus encephalitis [10], multiple sclerosis [11], Huntington’s [12] and Alzheimer’s disease [13].

Given the extensive evidence that TSPO is upregulated in neuroinflammation, there is a significant clinical interest in optimising TSPO PET imaging to better capture various facets of inflammation in human brain disorders [14, 15]. A number of radiotracers have thus been developed to visualize TSPO in the brain. One of the earliest TSPO PET tracers is [11C]PK11195, still routinely used in clinical imaging but hampered by the poor signal-to-noise ratio arising from its low binding affinity [16, 17]. To overcome this problem, numerous ‘second-generation’ TSPO PET tracers with increased affinity have been developed, including [11C]PBR28 [18]. However, the clinical use of these second-generation tracers is complicated by a single nucleotide polymorphism in the *Tspo* gene that alters its binding affinity in a proportion of humans. This necessitates genotyping of subjects along with excluding human participants who exhibit the “low affinity binder” status [19, 20]. Interestingly, this phenomenon has not been detected in rodents.

Although increased TSPO expression has historically been mostly associated with microglial activation, TSPO is now known to be expressed in almost all CNS cells including astrocytes, neurons and endothelial cells [21–27], all of which may also be involved in, or be affected by neuroinflammation [28]. Hence, to effectively utilise TSPO as a marker of neuroinflammation in general, as well as to ascertain its specificity in neurodegeneration, it is important to understand the contribution of these cell types toward the TSPO signal during various pathologies and therapeutic interventions [21]. To this end, we examined the expression and distribution of TSPO in a murine model of prion disease, the ME7 mouse. Prion diseases are a group of chronic transmissible neurodegenerative diseases, characterized by both progressive neuronal loss and neuroinflammation [29, 30]. Prion disease experimental models are robust and tractable laboratory tools to study these pathological processes [31, 32]. Using the ME7 mouse, we previously corroborated the ability of the selective colony stimulating factor receptor 1 (CSF1R) inhibitor, JNJ-40346527 (JNJ527), to significantly block microglial proliferation. This was demonstrated by the reduced binding of the first generation TSPO tracer, [3H]PK11195, using autoradiography [33]. Here, we extended these examinations further to (1) explore the sensitivity of the second-generation TSPO radiotracer [3H]PBR28 to detect neuroinflammation, and pharmacological modulation of microglia population by JNJ527, in the ME7 model, and (2) investigate the origin of the acquired TSPO signal changes in terms of their cellular composition.

## Methods

### Animals—experimental model of prion disease

C57BL/6J mice (*n* = 7–8/group) were housed under controlled environmental conditions (22 ± 1 °C and a 12-h light/12-h dark cycle) with free access to food and water. To induce prion disease, 10-week-old mice were anaesthetized with a ketamine/xylazine mixture (85 and 13 mg/kg), and 1 µl of either ME7-derived (ME7 animals) brain homogenate (10% w/v) or normal brain homogenate (NBH animals) was injected stereotaxically and bilaterally in the CA1 layer of dorsal hippocampus, at coordinates from Bregma: anteroposterior, − 2.0 mm; lateral, ± 1.7 mm; depth, − 1.6 mm [34]. JNJ527 was incorporated into mouse chow 12 weeks after prion infection as previously described by Olmos-Alonso [35], for a final dose of 30 mg/kg with an average daily ingestion of 5 g of food per mouse for 4 weeks. All animal studies were ethically reviewed and carried out in accordance with Animals (Scientific Procedures) Act 1986 and the GSK Policy on the Care, Welfare and Treatment of Animals.

### Tissue collection

Immediately after the treatment, at the age of 26 weeks, prion-infected and age-matched control (NBH) mice were terminally anaesthetized with an overdose of sodium pentobarbital and transcardially perfused with 0.9% saline. Whole brains were excised and divided into two hemispheres. One hemisphere was flash frozen in chilled isopentane (ca. − 35 °C) and stored at − 80 °C until required for autoradiography. The other hemisphere was placed in 4% w/v paraformaldehyde (PFA) for 24 h, followed by immersion in 30% w/v sucrose solution and stored at 4 °C until required for assessment by immunohistochemistry.

### Autoradiography

Brain hemispheres from normal brain homogenate (NBH) control group, ME7 and ME7 + JNJ527 mice (*n* = 7–8/group) were coronally cryosectioned at 20-µm thickness, mounted directly onto glass slides and processed for autoradiography as described previously [33].

Briefly, slides were incubated with 100 mM Tris–HCl, containing 1 nM [3H]PBR28 (specific activity 82.8 Ci/mmol, generously provided by Dr. David R. Owen, Imperial College) for 30 min, washed twice for 6 min in 100 mM Tris–HCl, rinse-dipped in dH_2_O and air dried. Non-specific binding was determined on adjacent sections in the presence of unlabelled PK11195 (20 µM; Sigma-Aldrich, #85532-75-8). Slides were exposed to tritium-sensitive film (Amersham Hyperfilm MP, GE Healthcare) in autoradiography cassettes together with a set of tritium standards ([3H] Microscale, American Radiolabelled Chemicals, #art-0123A) for 6 weeks. Quantitative analysis was performed using MCID image analyzer (Image Research, Canada), and the brain structures were identified using the mouse brain atlas of Franklin and Paxinos [36]. Cortex, hippocampus and thalamus regions of interest (ROI) were analysed by freehand drawing tools in two consecutive sections per brain (i.e., total and non-specific binding sections (NSB) were taken adjacently). Data are represented as individual mouse reading from the average of two sections from each ROI per animal.

### Histology

The remaining hemispheres from NBH (controls), ME7 and ME7 + JNJ527 mice (*n* = 7–8/group) were cut at 35 µm thickness using the HM 430 Sliding Microtome (Thermo Fisher Scientific).

Immunohistochemistry for TSPO (ab109497, Abcam) and triple immunofluorescence of TSPO (ab109497, Abcam), Iba1 a marker for both microglia and macrophages (ab5076, Abcam) and GFAP astrocytes (ab4674, Abcam); along with triple immunofluorescence of TSPO (ab109497, Abcam), NeuN for neurons (266006, Synaptic Systems) and CD31 for vascular endothelium (AF3628, R&D Systems) were performed to determine brain TSPO protein expression and TSPO cellular sources.

Free floating sections were incubated in citrate buffer pH = 8 at 80 °C for 30 min and 0.3% v/v H2O2 to block for endogenous peroxidase activity (only for bright field immunohistochemistry), and with 10% w/v milk and 0.3% v/v Triton X‐100 in TBS for 40 min. After rinses with tris buffered saline (TBS), sections were incubated overnight at 4 °C with rabbit anti‐TSPO antibody (1:10,000 in blocking solution, ab109497, Abcam) for bright field immunohistochemistry, and with a mix of primary antibodies for immunofluorescence (see Additional file 1: Table S1). After washes with TBS, sections were incubated with the appropriate biotinylated (Vector Labs) or Alexa-conjugated secondary antibodies (see Additional file 1: Table S1).

#### Immunohistochemistry

For bright field immunohistochemistry, following rinsing, the sections were incubated with Vectastain Elite ABC peroxidase kit (Vectors Labs) and developed with diaminobenzidine (DAB). Sections were mounted with DPX and images were captured at 40× magnification with photomicrographs captured using a Virtual Slide Microscope VS120 (Olympus Life Science). ROIs from the cortex, hippocampus and thalamus (3–4 sections/ROI) were analysed by measuring the percentage area covered by TSPO in 8640 µm × 8640 µm images using ImageJ (NIH, USA) and the average of an individual mouse from all available sections (3–4) from each region of interest per animal was represented.

#### Immunofluorescence

For immunofluorescence, sections were counterstained with 4′,6-diamidino-2-phenylindole (DAPI) and mounted with FluorSave™ mounting medium (345789-20, Calbiochem). Imaging was performed using an inverted spinning disc confocal microscope system (Nikon Eclipse T1). For relative quantification of immunofluorescence, one 2490 µm × 2490 µm photomicrographs containing series of ~ 10 µm deep Z stacks, corresponding to ~ 12 optical sections at 60× fields from the three fluorescence channels were captured from a dentate gyrus area of the hippocampus (approximately Bregma − 3.24 mm) per animal. For each photomicrograph, the total number of (i) Iba1+ cells (microglia/macrophages), (ii) GFAP+ cells (astrocytes), (iii) NeuN+ cells (neurons), (iv) TSPO+Iba1+ cells (microglial/macrophages cells expressing TSPO protein), (v) TSPO+GFAP+ cells (astrocytic cells expressing TSPO protein), (vi) TSPO+NeuN+ cells (neuronal cells expressing TSPO protein) and (vii) TSPO+CD31+ area (endothelial area expressing TSPO protein) were counted using NIS Elements Nikon software by thresholding, with DAPI stained nuclei as counterstain as follows. For the analysis of Iba1+ cells, GFAP+ cells and NeuN+ cells, two channel images of each field were analysed, the DAPI staining image was converted to a DAPI staining mask, and the specific staining image to a specific staining mask. A third mask corresponding to the nuclei of the immunostained cells was created using the combined image. Finally, immunostained cell nuclei were counted using the “Analyze Particles” tool. For the analysis of TSPO+Iba1+ cells, TSPO+GFAP+ cells and TSPO+NeuN+ cells, three channel images were analysed per each field and the final mask corresponded to the nuclei of the double immunostained cells.

## Statistics

Autoradiography data were analysed using a mixed-effects model ANOVA with ROI as within-subject factor and treatment group as between-subject factor followed by post hoc Sidak tests. The remaining data were analysed using a one-way ANOVA followed by post hoc Tukey tests*.* Quantitative data are expressed as mean ± standard error of the mean (SEM) and analysed with GraphPad Prism (version 8). *p* value < 0.05 was considered to be statistically significant.

## Results

### Increased [3H]PBR28 binding in ME7-prion mice and pharmacological attenuation by JNJ527

We previously demonstrated that the selective CSF1R inhibitor JNJ527 significantly blocked microglial proliferation in the ME7 mouse model of prion disease, and this has been detected by ex vivo autoradiography with the first generation TSPO ligand, [3H]PK11195 [33]. We repeated these observations here, and additionally demonstrated the increased sensitivity and specificity of the second-generation tracer, [3H]PBR28, to detect TSPO signal in this model. [3H]PBR28 showed a complete lack of non-specific binding when visually compared to [3H]PK11195 (Additional file 1: Fig. S1), and a larger relative increase in binding to the ME7 brains compared to NBH controls with [3H]PBR28 compared to [3H]PK11195 in cortex (~ twofold vs. 0.9-fold), hippocampus (~ 1.5-fold vs. 0.95-fold) and thalamus (~ threefold vs. 1.5-fold) (Additional file 1: Fig. S1).

**Fig. 1:**
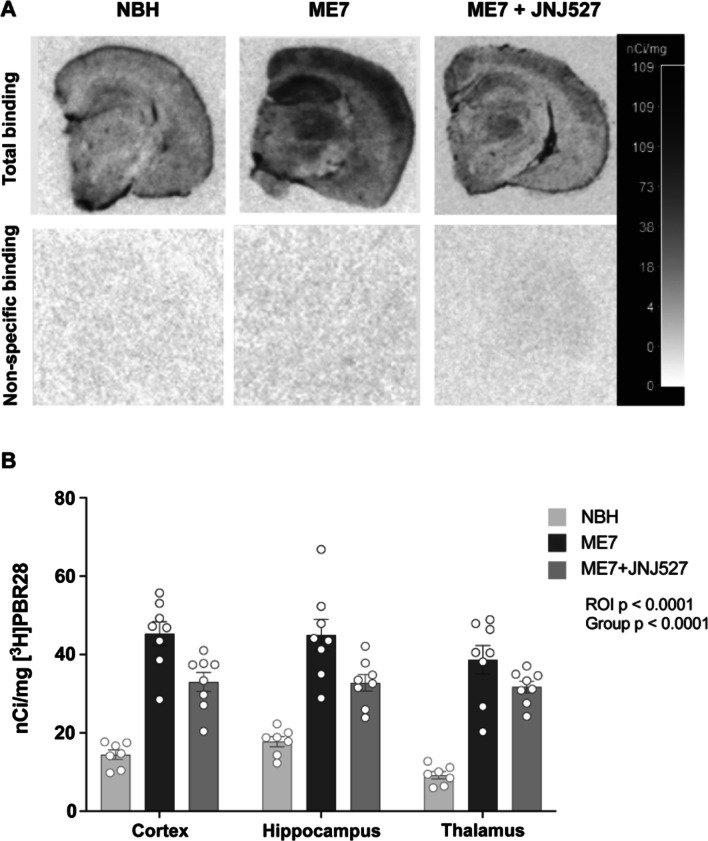
[3H]PBR28 binding in ME7-prion mice and pharmacological attenuation by JNJ527. **A** Representative autoradiographs of [3H]PBR28 in NBH (normal brain homogenate), ME7 and ME7 + JNJ527 mice brains (upper panels represent total signal and lower panels represent non-specific signal). **B** Mean signal intensity binding of [3H]PBR28 for cortex, hippocampus and thalamus from the three treatment groups. Each dot represents an individual mouse’s data from an average of two consecutive sections from each region of interest. Bars represent mean ± standard error of the mean. For ROI placement examples see Additional file 1: Fig. S2A. Statistical differences: significant effect of treatment group (*F* (2, 20) = 40.14, *p* < 0.0001) and ROI (*F* (1.998, 39.96) = 11.90, *p* < 0.0001) with no significant group × ROI interaction (*F* (4, 40) = 2.227, *p* = 0.0833). Data were analysed with a mixed-effects model ANOVA with ROI as within-subject factor and treatment group as between-subject factor followed by post hoc Sidak tests (*n* = 7–8/group)

Mixed-effects model ANOVA analysis of [3H]PBR28 autoradiography showed a significant effect of treatment group (*p* < 0.0001) and ROI (*p* < 0.0001) with no significant group × ROI interaction (*p* = 0.0833) (see Table 1A). [3H]PBR28 binding was significantly increased in the cortex, hippocampus and thalamus of ME7 vs. NBH mice, in line with previous findings [33, 37]. This increase was attenuated by the JNJ527 treatment in all areas (Fig. 1A, B), although the attenuation appears to be greater in the cortex and the hippocampus than in the thalamus (Fig. 1A, B).

**Table 1:** Significant main effect of treatment (NBH, ME7, ME7 + JNJ527) across the cortex, thalamus and hippocampal regions

(A) [3H]PBR28 autoradiography	
Mixed effects ANOVA factors	
Treatment_(NBH, ME7, ME7+JNJ527)_	*F* (2,20) = 40.14, *p* < 0.0001
ROI_(cortex, hippocampus, thalamus)_	*F* (1.998, 39.96) = 11.90, *p* < 0.0001
Treatment × ROI	*F* (4,40) = 2.227, *p* = 0.0833
(B) TSPO immunohistochemistry	
ROI	Main effect of treatment_(NBH, ME7, ME7+JNJ527)_
Cortex	*F* (2,20) = 8.110, *p* = 0.0026
Thalamus	*F* (2,19) = 15.72, *p* < 0.0001
CA1 hippocampus	*F* (2,20) = 14.63, *p* = 0.0001
CA3 hippocampus	*F* (2,20) = 8.509, *p* = 0.0021
Dentate gyrus	*F* (2,20) = 16.30, *p* < 0.0001
(C) Immunofluoresence in the dentate gyrus	
Marker	Main effect of treatment_(NBH, ME7, ME7+JNJ527)_
TSPO	*F* (2,14) = 3.473, *p* = 0.0596
Iba 1	*F* (2,18) = 2.606, *p* = 0.0003
GFAP	*F* (2,20) = 1.096, *p* = 0.0017
NeuN	*F* (2,16) = 8.395, *p* < 0.0001
(D) Immunofluoresence co-localization in the dentate gyrus	
Marker	Main effect of treatment_(NBH, ME7, ME7+JNJ527)_
TSPO+ Iba1+	*F* (2,19) = 16.34, *p* < 0.0001
TSPO+ GFAP+	*F* (2,20) = 10.84, *p* = 0.0012
TSPO+ NeuN+	*F* (2,16) = 34.55, *p* < 0.0001
TSPO+ CD31+	*F* (2,15) = 2.099, *p* = 0.1571

(A) [3H]PBR28 autoradiography. Mixed-effects repeated measures analysis (*F* tests and corresponding *p* values) showing a significant main effect of treatment group (normal brain homogenate NBH, ME7 prion infected, and ME7 prion infected treated with JNJ527), a significant main effect of region of interest (ROI; cortex, hippocampus and thalamus) but no significant interaction between the two factors. (B) TSPO immunohistochemistry. *F* tests and corresponding *p* values for an unpaired one-way ANOVA in the cortex, thalamus and several regions in the hippocampus: CA1, CA3, dentate gyrus (DG). (C) TSPO, Iba1, GFAP and NeuN immunofluorescence. *F* tests and corresponding *p* values of the effects of treatment (NBH, ME7 and ME7 + JNJ527) for each marker in the DG. (D) Co-localization immunofluorescence analysis of TSPO with Iba1, GFAP, NeuN and CD31. *F* tests and corresponding *p* values of the main effect of treatment (NBH, ME7, ME7 + JNJ527) for each marker (TSPO and Iba1, TSPO and GFAP, TSPO and NeuN, TSPO and CD31)

### Increased regional brain TSPO protein expression in ME7-prion mice and pharmacological attenuation by JNJ527 in the dentate gyrus of the hippocampus

**Fig. 2:**
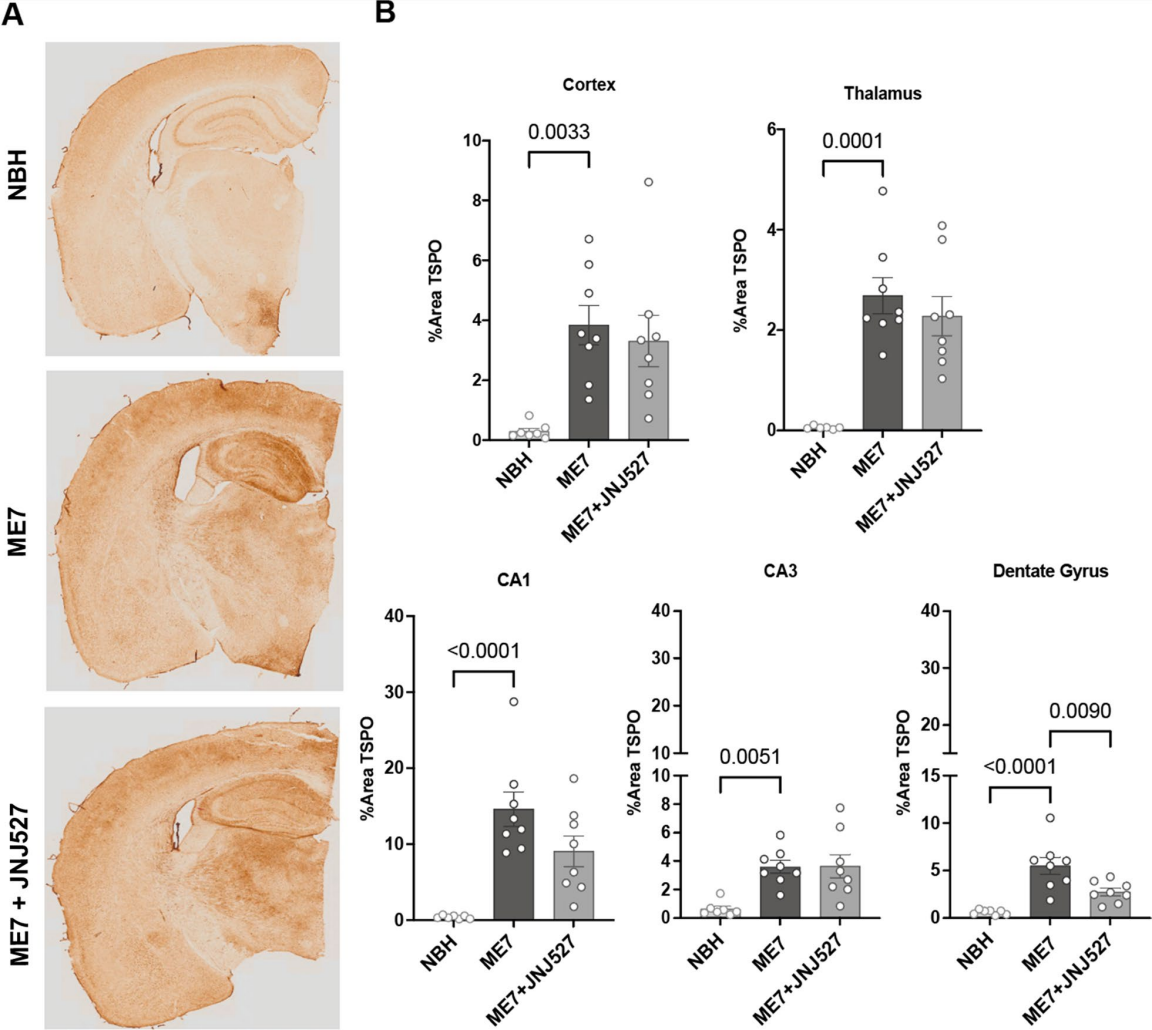
TSPO regional brain expression in ME7-prion mice and pharmacological attenuation by JNJ527. **A** Representative TSPO immunohistochemistry images of NBH, ME7 and ME7 + JNJ527 mouse brains. **B** Quantification of percentage area occupied by TSPO staining in brain regions from the three treatment groups. Each single dot represents an individual mouse’s data from the average of 3–4 sections from each region of interest per animal. Bars show mean ± standard error of the mean. For ROI placement examples see Additional file 1: Fig. S2B. Statistical differences: significant effect of treatment group in the cortical (*F* (2, 20) = 8.110, *p* = 0.0026), thalamic (*F* (2,19) = 15.72, *p* < 0.0001), and hippocampal regions [CA1 (*F* (2, 20) = 14.63, *p* = 0.0001), CA3 (*F* (2, 20) = 8.509, *p* = 0.0021) and the dentate gyrus (*F* (2, 20) = 16.30, *p* < 0.0001)] (Table 1). Data were analysed with a one-way ANOVA followed by post hoc Tukey tests (*n* = 7–8/group)

We next investigated whether the observed increase in [3H]PBR28 binding in the brains of ME7 mice was also associated with an increased expression of the TSPO protein. The contralateral hemisphere of each brain used for autoradiography was investigated for TSPO expression by immunohistochemistry. To this end, tissue sections containing regions of the cortex, thalamus and hippocampus were immunostained with an anti-TSPO antibody (Fig. 2A). Using thresholding analysis we detected a significant effect of treatment group in the cortical (*p* = 0.0026), thalamic (*p* < 0.0001), and hippocampal regions (CA1, *p* = 0.0001; CA3, *p* = 0.0021; and the dentate gyrus, *p* < 0.0001) (see Table 1B). Upon post hoc analysis we demonstrated that TSPO protein expression was significantly increased in ME7 vs. NBH in the cortical (*p* = 0.0033), thalamic (*p* < 0.0001), CA1 (*p* < 0.0001), CA3 (*p* = 0.0051) and dentate gyrus (*p* < 0.0001) (Fig. 2AB), confirming increased TSPO expression in ME7 mice compared to NBH across all ROIs. Of the regions studied, treatment with JNJ527 significantly reduced the TSPO protein expression in the dentate gyrus (*p* = 0.0090) with a trend toward a decrease in the CA1 (*p* = 0.09, Fig. 2B). Of note, we observed a higher degree of variability in these immunohistochemistry data than with autoradiography, which is commented on later in this manuscript.

### JNJ527 attenuates Iba1+ microglial and neuronal cell number but not GFAP+ astrocytes in the dentate gyrus of ME7-prion mice

**Fig. 3:**
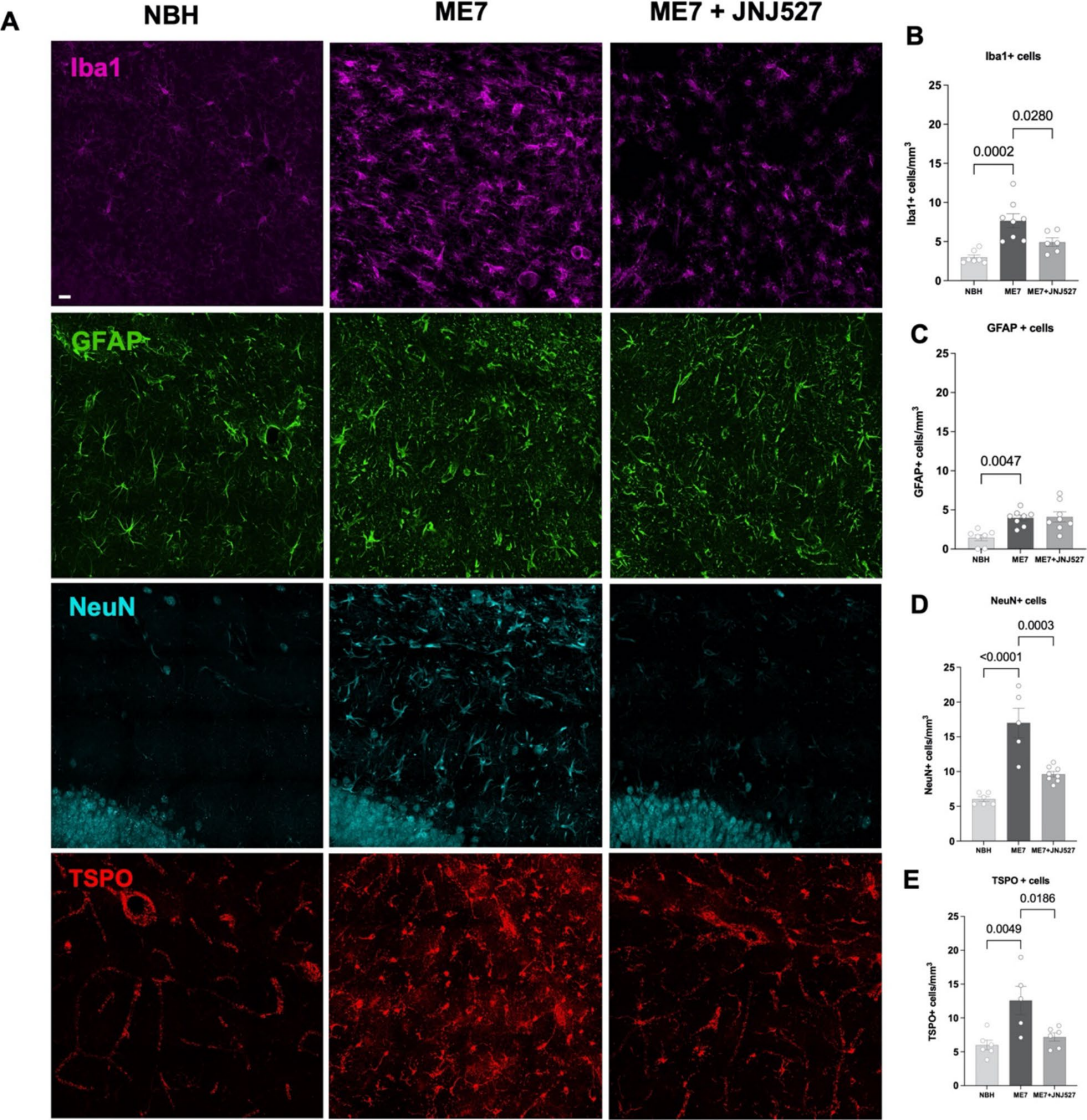
Microglia/macrophages, astrocytes, neurons and TSPO in the dentate gyrus of the hippocampus in NBH, ME7, ME7 + JNJ527 mice. **A** Confocal photomicrographs from dentate gyrus-immunostained sections. Microglia/macrophages, Iba1 (purple), astrocytes, GFAP (green), neurones, NeuN (cyan) and TSPO (red). **B** Cell counts expressed as Iba1+ cells, **C** GFAP+ cells, **D** NeuN+ cells, **E** TSPO+ cells from the three treatment groups (NBH, ME7 prion infected mice, and ME7+ treatment with JNJ527). Scale bar = 20 μm. Each single dot represents an individual mouse’s data from one section from each region of interest from an ROI in the dentate gyrus placed as in Additional file 1: Fig. S2B. Error bars represent mean ± standard error of the mean. Statistical differences: significant effect of treatment group on the number of Iba1+ cells (*F* (2, 18) = 2.606, *p* = 0.0003), GFAP+ cells (*F* (2,20) = 1.096, *p* = 0.0017) and NeuN+ cells (*F* (2, 16) = 8.395, *p* < 0.0001). Data were analysed with a one-way ANOVA followed by post hoc Tukey tests (*n* = 7–8/group)

Given the attenuation of TSPO expression by JNJ527 in the hippocampus, which appeared to be most robust in the dentate gyrus, we focused on this brain area. We first investigated the impact of JNJ527 induced CSF1R inhibition on the microglial, astrocytic and neuronal populations (Fig. 3). As expected, we detected an overall significant effect of treatment group on the number of Iba1+ cells (*p* = 0.0003, Fig. 3B), GFAP+ cells (*p* = 0.0017, Fig. 3C) and NeuN+ cells (*p* < 0.0001, Fig. 3D) (see Table 1C). In line with previous results [31, 33, 38, 39], upon post hoc analysis we corroborated the increase in the number of Iba1+ cells (*p* = 0.0002), GFAP+ cells (*p* = 0.0047) and NeuN+ cells (*p* < 0.0001) in ME7 vs. NBH. In addition, a significant reduction was induced by JNJ527, in Iba1+ cells (*p* = 0.0280) and in NeuN+ cells (*p* = 0.0003); however, there was no effect of JNJ527 on the number of GFAP+ cells (Fig. 3). We further confirmed a significant effect of treatment group on the number of TSPO-positive (TSPO+) cells (*p* = 0.0596, Fig. 3E) and upon post hoc analysis we corroborated the increased TSPO+ cells (*p* = 0.0049) in ME7 vs. NBH and a significant reduction induced by JNJ527 (*p* = 0.0186, Fig. 3E).

### Increased brain TSPO expression in Iba1+ microglia, GFAP+ astrocytes, neurons and endothelium in the ME7 mice and pharmacological attenuation by JNJ527 in Iba1+ microglia and neurons

**Fig. 4:**
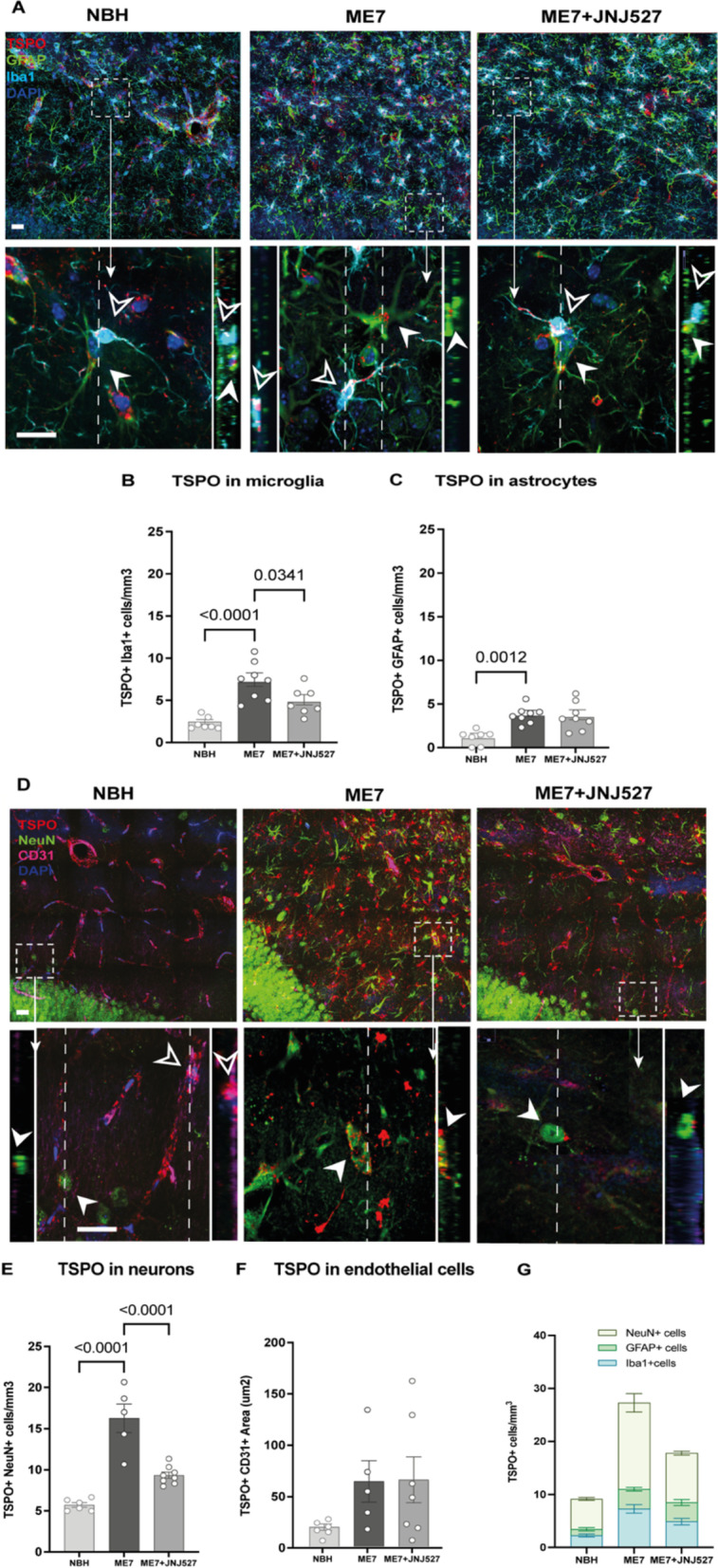
TSPO expression in microglia, astrocytes, neurons and endothelium in the dentate gyrus of the hippocampus in the ME7−prion mice. **A**, **D** Confocal photomicrographs from dentate gyrus-immunostained sections from NBH, ME7, ME7 + JNJ527 mice. **A** Microglia/macrophages, Iba1 (cyan), astrocytes, GFAP (green), and TSPO (red). **D** Neurons, NeuN (green), endothelial cells, CD31 (purple) and TSPO (red). Scale bar = 20 μm for top panels **A** and **D**. Magnifications at 50 μm showing TSPO (red) in astrocytic cells (green) and in microglia/macrophages (cyan) (bottom panel **A**) and TSPO (red) in neuronal cells (green) and in the vascular endothelium (purple) (bottom panel **D**) with their confocal lateral views showing co-localization of TSPO in the different cell types. Nuclear counterstaining was performed with DAPI (blue). For panel **A**: white arrowsheads: cells positive for GFAP and TSPO; empty arrowheads: cells positive for Iba1 and TSPO and for panel **D**: white arrowsheads: cells positive for NeuN and TSPO; empty arrowheads: cells positive for CD31 and TSPO. **B** Cell counts expressed as TSPO+Iba1+, **C** cell counts expressed as TSPO+GFAP+ cells, **E** cell counts expressed as TSPO+NeuN+, **F** area expressed as TSPO+CD31+ area and **G** representation of TSPO+Iba1+, TSPO+GFAP+ and TSPO+NeuN+ cells from the dentate gyrus from the three treatment groups. Each single dot represents an individual mouse’s data from one selected section from each region of interest from an ROI in the dentate gyrus placed as shown in Additional file 1: Fig. S2B. Error bars represent mean ± standard error of the mean. Statistical differences: significant differences in the number of TSPO+Iba1+ cells (*F* (2, 19) = 16.34, *p* < 0.0001), TSPO+GFAP+ cells (*F* (2, 20) = 10.84, *p* = 0.0012) and TSPO+NeuN+ cells (*F* (2, 16) = 34.55, *p* < 0.0001) but not in the TSPO+CD31+ area (*F* (2, 15) = 2.099, *p* = 0.1571). Data were analysed with a one-way ANOVA followed by post hoc Tukey tests (*n* = 7–8/group)

To investigate the extent to which the changes in TSPO expression reflect its expression in microglia, astrocytes, neurons or endothelial cells, we performed co-localization immunofluorescence. We examined the dentate gyrus of the hippocampus, as before, and measured microglial and astrocytic activation as well as an increase in the neuronal population induced by prion disease (Fig. 3). We identified four types of cells, based on the expression of TSPO and of specific markers: (i) TSPO positive that are also positive for Iba1 (TSPO+Iba1+) for microglia; (ii) TSPO positive that are also positive for GFAP (TSPO+GFAP+) for astrocytes; (iii) TSPO positive that are also positive for NeuN (TSPO+NeuN) for neurons; and iv) TSPO positive that are also positive for CD31 (TSPO+CD31) for endothelium (measured here as an area (mm^2^) rather than the number of cells). Co-localization analysis revealed a similar number of TSPO+Iba1+cells (2.26 ± 0.27) and TSPO+GFAP+cells (1.15 ± 0.31) in the control mice (NBH, Fig. 4A, first column) and a higher TSPO expression in neurons (5.72 ± 0.29) and area in endothelium (18.9 ± 2.9) (NBH, Fig. 4D, first column). Comparison between NBH controls and ME7 groups demonstrated significant increases in the number of TSPO+Iba1+ cells (*p* < 0.0001, Fig. 4B), TSPO+GFAP+ cells (*p* = 0.0012, Fig. 4C) and TSPO+NeuN+ cells (*p* < 0.0001, Fig. 4E) but not in the TSPO+CD31+ area (*p* = 0.1571, Fig. 4F) (see Table 1D). Post hoc analysis confirmed that ME7 mice showed a significant (~ 2.2-fold) increase in both the number of TSPO+Iba1+ cells (*p* < 0.0001, Fig. 4B) and TSPO+GFAP+ cells (*p* = 0.0012, Fig. 4C) compared to controls, along with a significant (~ 1.8-fold) increase in the number of TSPO+NeuN+ cells compared to controls (*p* < 0.0001, Fig. 4E) and an increase in the TSPO+CD31+ area [did not reach statistical significance (Fig. 4F)]. Treatment with JNJ527 significantly reduced the number of TSPO+Iba1+ cells (*p* = 0.0341, Fig. 4B) and TSPO+NeuN+ cells (*p* < 0.0001, Fig. 4E), but had no effect on the number of TSPO+GFAP+ cells (*p* = 0.9648, Fig. 4C) or the TSPO+CD31+ area (Fig. 4F) compared to untreated ME7-prion mice.

## Discussion

Imaging neuroinflammation in neurodegeneration with TSPO PET tracers to monitor response in clinical trials of novel therapies has been a major focus of interest in past years. However, no clear consensus has yet been reached regarding the cellular and functional interpretation of the TSPO PET signal. This is most likely driven by the heterogeneity of TSPO expression in the brain, its cellular sources, as well as binding affinity differences of TSPO PET imaging agents. Consequently, we sought to perform focused studies to address some of these themes which are now reported in this manuscript.

The aims of the current study were (1) to evaluate the potential of the second-generation TSPO radiotracer PBR28 to measure neuroinflammation by ex vivo autoradiography in a mouse model of chronic prion-induced neurodegeneration (ME7) with and without pharmacological intervention with a selective CSF1R inhibitor; and (2) to investigate the origin of TSPO signal changes in terms of cellular sources and regional distribution. CSF1R inhibitor JNJ527 is a tool compound that was previously shown to attenuate microglia proliferation and provide therapeutic action in models of neurodegeneration including ME7 prion mice and in the P301S mouse tauopathy model [33].

We first performed high resolution ex vivo TSPO autoradiography with [3H]PBR28 which confirmed the increased binding, and, therefore, expression of TSPO, in the cortical, hippocampal and thalamic brain regions of ME7 mice compared to NBH controls. This demonstration of increased TSPO in experimental prion disease aligns with ours and others’ earlier findings using the first generation TSPO radiotracer [3H]PK11195 [33, 37]. Furthermore, as expected, pharmacological inhibition of CSF1R with JNJ527 attenuated the increased [3H]PBR28 binding (Fig. 1) in multiple brain areas. We also show that [3H]PBR28 is superior to [3H]PK11195 with regard to the signal to noise ratio (Additional file 1: Fig. S1) and the observed effect size of pharmacological intervention (Additional file 1: Table S2). These findings are consistent with clinical data indicating that [3H]PBR28 appears to be more sensitive than [3H]PK11195 for detecting TSPO in chronic neuroinflammation in human tissue [40]. Our results thus confirm the superiority of second-generation TSPO autoradiography to detect neuroinflammation and its modulation in neurodegeneration.

To study the cellular composition of TSPO signal we used immunohistochemistry. The analysis of immunohistochemically stained sections confirmed an overall increase in TSPO expression in the cortex, thalamus and hippocampus in ME7 mice, compared to NBH controls. Unlike in ex vivo autoradiography; however, JNJ527-treated animals’ brains showed only a trend toward the expected TSPO signal decrease in most of the examined areas, with the exception of the hippocampal dentate gyrus, where the signal was significantly lower. This discrepancy could be explained by the greater variability of immunohistochemistry results compared to ex vivo autoradiography. The increased variability is likely resulting from the non-linear nature of chromogenic (diaminobenzedine) immunohistochemistry signal used here which, unlike in immunofluorescence, is not directly related to the underlying antigen because of the enzymatic amplification steps necessary to produce and visualise coloured precipitate in the tissue [41]. In addition, further variability may be introduced during the staining process itself, as the signal of the background is heavily influenced by the length of time the final enzymatic reaction is allowed to proceed. Consequently, the quantification of DAB-based signal is challenging; although we tried to circumvent this confound by performing a threshold analysis, rather than direct signal quantification, our immunohistochemistry measurements are likely to be less sensitive and not as representative of the underlying ground truth, compared to autoradiography.

The hippocampus was one area of the brain, where the TSPO signal was robustly increased in ME7 mice, seen on both autoradiography and immunohistochemistry, and this increase was attenuated by JNJ527. Specifically, the dentate gyrus, a region with abundance of glial cells and an enriched neuronal population, showed a robust increase in TSPO signal in ME7 mice, which was significantly reduced by treatment with JNJ527 (Fig. 2). For this reason, this sub-area of the hippocampus was chosen to further examine the cellular specificity of the TSPO signal. The dentate gyrus is also one of the two principal neurogenic niches, where stem cells can differentiate into neuroblasts that have the potential to migrate to sites of injury in a potential compensatory neural repair role [42, 43]. Our interest in the dentate gyrus was furthered by earlier discoveries that aberrant increased neurogenesis takes place during some neurodegenerative sequelae including that in experimental prion disease [31]. Given that more recent evidence confirms TSPO expression in neurons [44], along with the established presence in the glial and endothelial cells [26, 45], we were keen to examine the contribution of neurons, glia and endothelial cells to TSPO signal in ME7 mice.

Prior to investigating the cellular diversity of TSPO, we examined the response of different cell types individually to ME7 prion infection. We detected an increased in GFAP+ astrocytes, Iba1+ microglia and neurons in the dentate gyrus (Fig. 3). Gliosis is consistent with the presence of neuroinflammation [46]. Microgliosis and astrocytosis were previously described in the dentate gyrus area of the hippocampus of prion disease mice [32, 38, 39]. The observed increase in neurons indirectly supports the aforementioned evidence of increased neurogenesis and potentially reflects the aberrant placement of neurons [47], although we did not measure neurogenesis directly here as this was beyond the scope of the study.

We next examined the relative distribution of TSPO by colocalization of TSPO signal with the markers of different cell types: Iba1 for microglia/macrophages, GFAP for astrocytes, NeuN for neurons and CD31 for endothelial cells. We first confirmed the presence of TSPO in all those cell types in the control (NBH) mice: ~ 25% of the total cells were TSPO+Iba1+ cells, ~ 13% TSPO+GFAP+ cells and ~ 63% TSPO+NeuN+ cells in the control mice (NBH, Fig. 4G). Following ME7 infection, there was a significant increase in the number of all examined TSPO+ cells. The increases were more than twofold in Iba1+ microglia and GFAP+ astrocytes and ~ 1.8-fold increase in neurons and an increase in the endothelium area that did not reach statistical significance (Fig. 4F). This finding supports the notion that when visualising neuroinflammation by TSPO imaging, one should consider the multi-cellular nature of the signal increase. Indeed, our previous study examining cellular sources of TSPO in a rodent model of acute neuroinflammation, the LPS-challenged rat, also found a similar diversity of source of TSPO signal which was present in all examined cells at baseline, but then increased significantly in astrocytes and microglia upon inflammatory insult [21]. Similarly, others have found that TSPO positive microglia, astrocytes and endothelium are all increased in models of Alzheimer’s-like neurodegeneration [48–51] and in postmortem human Alzheimer’s disease brains [27].

The relatively novel finding here was of the increase in TSPO positive neuronal cells in ME7 mice brains. Given the finding of increased neurons in the dentate gyrus, this is unsurprising—if there are more neurons, and neurons contain TSPO, then it is expected that TSPO signal will increase. However, it is also possible that TSPO is functionally upregulated and participates in either the proliferation or redistribution of neurons in ME7 mice. Studies have demonstrated the presence of TSPO in immature neurons, as well as in differentiating/regenerating neurons [23, 24, 52]. TSPO is involved in the mitochondrial intermembrane transport of cholesterol which is the necessary precursor for the synthesis of neurosteroids [53–56], and both cholesterol and neurosteroids are important for neuronal regeneration [57]. Neurosteroids are also thought to be associated with promoting adult neurogenesis, alongside their other proposed roles of modulating synaptic transmission and neuronal network formation [58]. To this end, neurosteroids, together with their synthetic analogues, as well as TSPO ligands, are being investigated as potential therapeutic and neuroprotective agents [59–62]. All this, therefore, points toward a plausible role for modulatory function of TSPO in prion neurodegeneration and neurogenesis.

Examination of the effect of JNJ527 treatment on cells in the dentate gyrus revealed a significant reduction of the number of Iba1+ microglia/macrophages (*p* = 0.0280) and neurons (*p* = 0.0003), (Fig. 3B, D), but not of GFAP+ astrocytes and endothelium; this was matched by the observed reduction in the number of TSPO positive Iba1 positive microglia/macrophages (*p* = 0.0341) and neurons (*p* < 0.0001) (Fig. 4B, E). As CSF1R is thought to be expressed exclusively by microglia [34, 63, 64] and its inhibition reduces their proliferation, it is unsurprising that Iba1+ microglia/macrophages were also reduced by this treatment. However, CSF1R is not known to be expressed in neurons [38]. Nevertheless, the decrease in neuronal cells is in line with our earlier observations that pharmacological modulation of microglia with another CSF1R inhibitor, GW2580 was able to reduce neuronal population and aberrant neurogenesis and restore the normal neuronal differentiation [38]. Given that an increase in hippocampal neurogenesis was previously shown to correlate with the expansion of the microglia population [31] the results thus far corroborate the suggested intercellular communication occurring between the neurons and microglia in the dentate gyrus of the hippocampus during prion induced neurodegeneration.

In summary, our findings show that [3H]PBR28 autoradiography is an important translational tool for detecting and quantifying regional neuroinflammation both at baseline and following pharmacological intervention. In addition, TSPO overexpression in the ME7 mouse model of prion disease appears to be driven by a number of cell types: microglia/macrophages, astrocytes, neurons and endothelial cells. Inhibition of the number of Iba1+ microglial cells by JNJ527 induced a decrease in TSPO in both the Iba1+ microglia/macrophages and neurons as a consequence of the decrease in the neuronal and Iba1+ microglial population. Further work is now warranted to explore the nature of the connection between microglia/macrophages and neurons in the neuroinflammatory and neurodegenerative processes.

Researchers utilising TSPO to image neuroinflammation will need to consider the effect of multiple cell types contributing to the neuroinflammatory aspect of the disease itself and/or the effect of drug treatment when interpreting findings. This study also highlights the importance of coupling tissue autoradiography with relevant histology methods to define and understand the biological/cellular framework underpinning the neuroinflammatory processes which may differ between neurodegenerative diseases and will add to our overall interpretation and understanding of image data utilising TSPO PET agents.

## Supplementary Information

**Additional file 1: Figure S1.** Autoradiography comparison of [3H]PBR28 and [3H]PK11195 in ME7-prion mice and pharmacological attenuation by JNJ527. **Figure S2.** Example region of interest placement in autoradiography and immunohistochemical analyses. Examples of region of interest (ROI) placement in A) autoradiography analyses and B) immunohistochemical analyses. Placement of ROIs in the immunofluorescence analyses were in the same ROI for the dentate gyrus as shown in B. **Table S1.** Primary and secondary antibodies used for immunofluorescence. **Table S2.** Effect sizes for [3H]PK11195 and [3H]PBR28 autoradiography. **Appendix S1.** NIMA members during the sample collection and data analysis period for the BIODEP Study.

## Data Availability

The data sets generated during and/or analysed during the current study are available from the corresponding author on reasonable request.

## References

[1] Kwon HS, Koh S-H (2020). Neuroinflammation in neurodegenerative disorders: the roles of microglia and astrocytes. Transl Neurodegener.

[2] Banati RB, Middleton RJ, Chan R, Hatty CR, Wai-Ying Kam W, Quin C (2014). Positron emission tomography and functional characterization of a complete PBR/TSPO knockout. Nat Commun.

[3] Middleton RJ, Liu G-J, Banati RB (2015). Guwiyang Wurra–‘Fire Mouse’: a global gene knockout model for TSPO/PBR drug development, loss-of-function and mechanisms of compensation studies. Biochem Soc Trans.

[4] Tu LN, Morohaku K, Manna PR, Pelton SH, Butler WR, Stocco DM (2014). Peripheral benzodiazepine receptor/translocator protein global knock-out mice are viable with no effects on steroid hormone biosynthesis. J Biol Chem.

[5] Wang H, Zhai K, Xue Y, Yang J, Yang Q, Fu Y (2016). Global deletion of TSPO does not affect the viability and gene expression profile. PLoS ONE.

[6] Papadopoulos V, Baraldi M, Guilarte TR, Knudsen TB, Lacapère J-J, Lindemann P (2006). Translocator protein (18kDa): new nomenclature for the peripheral-type benzodiazepine receptor based on its structure and molecular function. Trends Pharmacol Sci.

[7] Rupprecht R, Papadopoulos V, Rammes G, Baghai TC, Fan J, Akula N (2010). Translocator protein (18 kDa) (TSPO) as a therapeutic target for neurological and psychiatric disorders. Nat Rev Drug Discov.

[8] Betlazar C, Middleton RJ, Banati R, Liu G-J (2020). The translocator protein (TSPO) in mitochondrial bioenergetics and immune processes. Cells.

[9] Kominsky DJ, Campbell EL, Colgan SP (2010). Metabolic shifts in immunity and inflammation. J Immunol.

[10] Vera JH, Guo Q, Cole JH, Boasso A, Greathead L, Kelleher P (2016). Neuroinflammation in treated HIV-positive individuals: a TSPO PET study. Neurology.

[11] Banati RB, Newcombe J, Gunn RN, Cagnin A, Turkheimer F, Heppner F (2000). The peripheral benzodiazepine binding site in the brain in multiple sclerosis. Brain.

[12] Meßmer K, Reynolds GP (1998). Increased peripheral benzodiazepine binding sites in the brain of patients with Huntington’s disease. Neurosci Lett.

[13] Cagnin A, Brooks DJ, Kennedy AM, Gunn RN, Myers R, Turkheimer FE (2001). In-vivo measurement of activated microglia in dementia. Lancet.

[14] Zhang L, Hu K, Shao T, Hou L, Zhang S, Ye W (2021). Recent developments on PET radiotracers for TSPO and their applications in neuroimaging. Acta Pharm Sin B.

[15] Werry EL, Bright FM, Piguet O, Ittner LM, Halliday GM, Hodges JR (2019). Recent developments in TSPO PET imaging as a biomarker of neuroinflammation in neurodegenerative disorders. Int J Mol Sci.

[16] Turkheimer FE, Rizzo G, Bloomfield PS, Howes O, Zanotti-Fregonara P, Bertoldo A (2015). The methodology of TSPO imaging with positron emission tomography. Biochem Soc Trans.

[17] Cumming P, Burgher B, Patkar O, Breakspear M, Vasdev N, Thomas P (2018). Sifting through the surfeit of neuroinflammation tracers. J Cereb Blood Flow Metab.

[18] Alam MM, Lee J, Lee S-Y (2010). Recent progress in the development of TSPO PET ligands for neuroinflammation imaging in neurological diseases. Nucl Med Mol Imaging.

[19] Owen DR, Yeo AJ, Gunn RN, Song K, Wadsworth G, Lewis A (2012). An 18-kDa translocator protein (TSPO) polymorphism explains differences in binding affinity of the PET radioligand PBR28. J Cereb Blood Flow Metab.

[20] Mizrahi R, Rusjan PM, Kennedy J, Pollock B, Mulsant B, Suridjan I (2012). Translocator protein (18 kDa) polymorphism (rs6971) explains in-vivo brain binding affinity of the PET radioligand [18 F]-FEPPA. J Cereb Blood Flow Metab.

[21] Vicente-Rodríguez M, Singh N, Turkheimer F, Peris-Yague A, Randall K, Veronese M (2021). Resolving the cellular specificity of TSPO imaging in a rat model of peripherally-induced neuroinflammation. Brain Behav Immun.

[22] Nutma E, Ceyzériat K, Amor S, Tsartsalis S, Millet P, Owen DR (2021). Cellular sources of TSPO expression in healthy and diseased brain. Eur J Nucl Med Mol Imaging.

[23] Girard C, Liu S, Adams D, Lacroix C, Sinéus M, Boucher C (2012). Axonal regeneration and neuroinflammation: roles for the translocator protein 18 kDa. J Neuroendocrinol.

[24] Mills CD, Bitler JL, Woolf CJ (2005). Role of the peripheral benzodiazepine receptor in sensory neuron regeneration. Mol Cell Neurosci.

[25] Betlazar C, Harrison-Brown M, Middleton RJ, Banati R, Liu GJ (2018). Cellular sources and regional variations in the expression of the neuroinflammatory marker translocator protein (TSPO) in the normal brain. Int J Mol Sci.

[26] Pannell M, Economopoulos V, Wilson TC, Kersemans V, Isenegger PG, Larkin JR (2020). Imaging of translocator protein upregulation is selective for pro-inflammatory polarized astrocytes and microglia. Glia.

[27] Gui Y, Marks JD, Das S, Hyman BT, Serrano-Pozo A (2020). Characterization of the 18 kDa translocator protein (TSPO) expression in post-mortem normal and Alzheimer’s disease brains. Brain Pathol.

[28] Carson MJ, Thrash JC, Walter B (2006). The cellular response in neuroinflammation: the role of leukocytes, microglia and astrocytes in neuronal death and survival. Clin Neurosci Res.

[29] Masters CL, Richardson EP (1978). Subacute spongiform encephalopathy (Creutzfeldt-Jakob disease). Brain.

[30] Williams A, Lucassen PJ, Ritchie D, Bruce M (1997). PrP deposition, microglial activation, and neuronal apoptosis in murine scrapie. Exp Neurol.

[31] Gomez-Nicola D, Suzzi S, Vargas-Caballero M, Fransen NL, Al-Malki H, Cebrian-Silla A (2014). Temporal dynamics of hippocampal neurogenesis in chronic neurodegeneration. Brain.

[32] Gomez-Nicola D, Perry VH (2015). Microglial dynamics and role in the healthy and diseased brain. Neurosci.

[33] Mancuso R, Fryatt G, Cleal M, Obst J, Pipi E, Monzón-Sandoval J (2019). CSF1R inhibitor JNJ-40346527 attenuates microglial proliferation and neurodegeneration in P301S mice. Brain.

[34] Gomez-Nicola D, Fransen NL, Suzzi S, Perry VH (2013). Regulation of microglial proliferation during chronic neurodegeneration. J Neurosci.

[35] Olmos-Alonso A, Schetters STT, Sri S, Askew K, Mancuso R, Vargas-Caballero M (2016). Pharmacological targeting of CSF1R inhibits microglial proliferation and prevents the progression of Alzheimer’s-like pathology. Brain.

[36] Franklin KBJ, Paxinos GP (1997). The mouse brain in stereotaxic coordinates.

[37] Song P-J, Barc C, Arlicot N, Guilloteau D, Bernard S, Sarradin P (2010). Evaluation of prion deposits and microglial activation in scrapie-infected mice using molecular imaging probes. Mol Imaging Biol.

[38] De Lucia C, Rinchon A, Olmos-Alonso A, Riecken K, Fehse B, Boche D (2016). Microglia regulate hippocampal neurogenesis during chronic neurodegeneration. Brain Behav Immun.

[39] Asuni AA, Gray B, Bailey J, Skipp P, Perry VH, O’Connor V (2014). Analysis of the hippocampal proteome in ME7 prion disease reveals a predominant astrocytic signature and highlights the brain-restricted production of clusterin in chronic neurodegeneration. J Biol Chem.

[40] Nutma E, Stephenson JA, Gorter RP, de Bruin J, Boucherie DM, Donat CK (2019). A quantitative neuropathological assessment of translocator protein expression in multiple sclerosis. Brain.

[41] van der Loos CM (2008). Multiple immunoenzyme staining: methods and visualizations for the observation with spectral imaging. J Histochem Cytochem.

[42] Keller D, Erö C, Markram H (2018). Cell densities in the mouse brain: a systematic review. Front Neuroanat.

[43] Kesner RP (2013). An analysis of the dentate gyrus function. Behav Brain Res.

[44] Notter T, Schalbetter SM, Clifton NE, Mattei D, Richetto J, Thomas K (2021). Neuronal activity increases translocator protein (TSPO) levels. Mol Psychiatry.

[45] Betlazar C, Harrison-Brown M, Middleton RJ, Banati R, Liu GJ (2018). Cellular sources and regional variations in the expression of the neuroinflammatory marker translocator protein (TSPO) in the normal brain. Int J Mol Sci.

[46] Bernaus A, Blanco S, Sevilla A (2020). Glia crosstalk in neuroinflammatory diseases. Front Cell Neurosci.

[47] Winner B, Kohl Z, Gage FH (2011). Neurodegenerative disease and adult neurogenesis. Eur J Neurosci.

[48] Ji B, Maeda J, Sawada M, Ono M, Okauchi T, Inaji M (2008). Imaging of peripheral benzodiazepine receptor expression as biomarkers of detrimental versus beneficial glial responses in mouse models of Alzheimer’s and other CNS pathologies. J Neurosci.

[49] Liu B, Le KX, Park M-A, Wang S, Belanger AP, Dubey S (2015). In vivo detection of age- and disease-related increases in neuroinflammation by 18 F-GE180 TSPO MicroPET imaging in wild-type and Alzheimer’s transgenic mice. J Neurosci.

[50] Tournier BB, Tsartsalis S, Rigaud D, Fossey C, Cailly T, Fabis F (2019). TSPO and amyloid deposits in sub-regions of the hippocampus in the 3xTgAD mouse model of Alzheimer’s disease. Neurobiol Dis.

[51] Cosenza-Nashat M, Zhao ML, Suh HS, Morgan J, Natividad R, Morgello S (2009). Expression of the translocator protein of 18 kDa by microglia, macrophages and astrocytes based on immunohistochemical localization in abnormal human brain. Neuropathol Appl Neurobiol.

[52] Varga B, Markó K, Hádinger N, Jelitai M, Demeter K, Tihanyi K (2009). Translocator protein (TSPO 18 kDa) is expressed by neural stem and neuronal precursor cells. Neurosci Lett.

[53] Ferzaz B, Brault E, Bourliaud G, Robert J-P, Poughon G, Claustre Y (2002). SSR180575 (7-chloro-N, N,5-trimethyl-4-oxo-3-phenyl-3,5-dihydro-4H-pyridazino[4,5-b]indole-1-acetamide), a peripheral benzodiazepine receptor ligand, promotes neuronal survival and repair. J Pharmacol Exp Ther.

[54] Korneyev A, Pan BS, Polo A, Romeo E, Guidotti A, Costa E (1993). Stimulation of brain pregnenolone synthesis by mitochondrial diazepam binding inhibitor receptor ligands in vivo. J Neurochem.

[55] Lacor P, Gandolfo P, Tonon M-C, Brault E, Dalibert I, Schumacher M (1999). Regulation of the expression of peripheral benzodiazepine receptors and their endogenous ligands during rat sciatic nerve degeneration and regeneration: a role for PBR in neurosteroidogenesis. Brain Res.

[56] Papadopoulos V, Amri H, Boujrad N, Cascio C, Culty M, Garnier M (1997). Peripheral benzodiazepine receptor in cholesterol transport and steroidogenesis. Steroids.

[57] Schumacher M, Robel P, Baulieu E-E (1996). Development and regeneration of the nervous system: a role for neurosteroids (part 1 of 2). Dev Neurosci.

[58] Tozzi A, Bellingacci L, Pettorossi VE (2020). Rapid estrogenic and androgenic neurosteroids effects in the induction of long-term synaptic changes: implication for early memory formation. Front Neurosci.

[59] Lloyd-Evans E, Waller-Evans H (2020). Biosynthesis and signalling functions of central and peripheral nervous system neurosteroids in health and disease. Essays Biochem.

[60] Reddy DS, Estes WA (2016). Clinical potential of neurosteroids for CNS disorders. Trends Pharmacol Sci.

[61] Lee Y, Park Y, Nam H, Lee J-W, Yu S-W (2020). Translocator protein (TSPO): the new story of the old protein in neuroinflammation. BMB Rep.

[62] Ratner MH, Kumaresan V, Farb DH (2019). Neurosteroid actions in memory and neurologic/neuropsychiatric disorders. Front Endocrinol (Lausanne).

[63] Erblich B, Zhu L, Etgen AM, Dobrenis K, Pollard JW (2011). Absence of colony stimulation factor-1 receptor results in loss of microglia, disrupted brain development and olfactory deficits. PLoS ONE.

[64] Sasmono RT, Oceandy D, Pollard JW, Tong W, Pavli P, Wainwright BJ (2003). A macrophage colony-stimulating factor receptor–green fluorescent protein transgene is expressed throughout the mononuclear phagocyte system of the mouse. Blood.

